# Optimized facial landmark modeling with medical aesthetic constraints by a multi-objective genetic algorithm

**DOI:** 10.3389/fncom.2026.1705259

**Published:** 2026-02-25

**Authors:** Yuan Ye, Gangxing Yan, Di Wen, Meijun Tan

**Affiliations:** 1 Department of Plastic and Cosmetic Surgery, Guangdong Women and Children Hospital, Guangzhou, China; 2 Faculty of Data Science, City University of Macau, Taipa, Macao SAR, China

**Keywords:** facial beauty assessment, genetic algorithm, machine learning, medical aesthetics, performance evaluation

## Abstract

“Facial Beauty” is not an absolute physical attribute but a subjective social and cultural construct. Facial beauty assessment is an interdisciplinary field that integrates computer vision and medical aesthetics (MAs) to quantify personal judgment regarding facial attractiveness. In this study, the beauty assessment we adopted was based on the scores given by plastic surgeons; this method is more professional and is supported by a theoretical basis. We derived a set of MA features that encompass global traits, local details, and curvature aspects from established aesthetic principles. Incorporating these features enhances predictive accuracy in facial beauty. Furthermore, we propose a feature selection algorithm with aesthetic-driven initialization embedded in a multi-objective evolutionary framework. Additionally, we introduce an MA facial landmark model that provides explicit annotation of bilateral zygomatic, orbital, and nasal points for precise attractiveness scoring. Experimental results on the South China University of Technology-Facial Beauty Perception (SCUT-FBP) and SCUT-FBP5500 datasets and the Chicago Face Dataset demonstrate superior performance (Pearson’s correlation coefficient = 0.8216, mean absolute error = 0.2638, and root mean square error = 0.3743) over state-of-the-art methods, validating its clinical relevance. This study provides a practical tool for beauty evaluation, where the selected features align with professional judgments, enabling transparent and explainable outcomes in both clinical and cosmetic applications.

## 1 Introduction

Accurate analysis of facial features is crucial in medical aesthetics (MAs) for targeted interventions. The human face, as a primary determinant of physical appearance, conveys substantial geometric information—including the positions, dimensions, and contours of facial features and their interrelationships—that underpins the assessment of attractiveness. Factors such as the spacing between the eyes, dimensions of the nose (including its height and length), the size and placement of the mouth, and dental alignment regularity (Tomášik et al., 2024) significantly influence attractiveness judgments. Even minor variations in these traits can dramatically affect overall facial attractiveness. In cosmetic surgery, surgeons tailor their strategies to the unique facial attributes of each patient to optimize symmetry and allure.

Multiple machine-learning methods (Lamassoure et al., 2021; Sable, 2021; Thomas et al., 2020) have been used to detect facial landmarks and features for aesthetic evaluation. Advancements in computer vision and image analysis have enabled deep neural networks to achieve robust estimation power in facial attractiveness assessment (Bae et al., 2024; Boukhari et al., 2023; Gan et al., 2023; Peng et al., 2024). However, the limited interpretability of deep-learning models impedes their uptake in clinical aesthetics and other fields that require accountability and transparency. Conventional approaches that rely on linear geometric features, such as absolute distances, angles, and proportional parameters, fail to model the complex morphological relationships critical for multidimensional facial analysis.

To address these limitations, we introduce a novel MA feature system validated by six domain specialists. Our hierarchical representation framework captures subtle morphological patterns beyond traditional linear measurements by measuring complex shape traits such as contour curvatures and eyelid inclinations. In this study, we curated a 135-dimensional feature set by integrating existing geometric features from the literature with 20 novel descriptors endorsed by 6 clinical experts (inter-rater reliability ICC = 0.87). A cascaded regression algorithm was developed to train the MA landmark model by efficiently incorporating all extracted features. This approach demonstrated greater computational efficiency compared with deep-learning methods, particularly when training data are limited.

Identifying discriminative features from high-dimensional spaces remains a persistent challenge in facial beauty assessment. Genetic algorithms (GAs), inspired by natural selection and genetic mechanisms, deliver robust exploration and flexible tuning. To leverage clinical expertise without compromising algorithmic efficiency, we propose an aesthetics-driven genetic algorithm scheme for feature selection. In the initialization phase, we infused prior MA knowledge by seeding the population with domain-specific features derived from clinical guidelines. This strategy ensures that the optimization process explicitly favors biologically meaningful attributes. We implemented a directional mutation strategy to limit variability in clinician-defined attributes, thus preserving core aesthetic parameters critical for clinical interpretation. This approach balances computational efficiency with clinical relevance and presents an effective avenue for real-world applications in facial aesthetics. We evaluated the proposed facial landmark model and feature selection approach across the South China University of Technology-Facial Beauty Perception (SCUT-FBP) and SCUT-FBP5500 datasets plus Chicago Face Dataset (CFD) portraits.

MA features are specifically designed for the clinical assessment of facial beauty. Unlike traditional geometric descriptors, these domain-specific features precisely captured nuanced shape differences across facial subunits. Extensive comparisons with baseline methods on multiple datasets show that our new descriptor pool boosts predictive accuracy. Specifically, our features showed superior ability to detect biologically relevant details, such as contour curvatures and symmetry metrics, which are critical for clinical interpretation. The experimental results confirmed that integrating these features markedly elevates the predictive performance of facial beauty models, underscoring their utility in precision aesthetic medicine.

Furthermore, our method integrates prior MA constraints with a multi-objective GA for facial beauty feature selection. By eliminating superfluous geometric details, we obtained a facial beauty feature set based on prior MA knowledge. Compared with other feature selection techniques, our method exhibited improved speed and accuracy. The proposed facial beauty evaluation model leverages prior MA knowledge and innovatively transforms the analytical criteria for plastic surgery into landmark spatial constraints. The optimized MA facial landmark model delivers top-tier outcomes in clinical attractiveness tests while requiring fewer computational resources.

## 2 Related work

### 2.1 Feature selection methods

Effective feature selection underpins the evaluation of facial beauty. Facial images contain large volumes of data, including geometric, texture, and color features. However, not all features are equally informative in facial beauty assessment. Redundant or irrelevant features can degrade model performance and lead to overfitting. Consequently, selecting the most discriminative feature subset from numerous facial features is essential for optimizing model accuracy. Traditional feature selection methods are typically classified into three categories: filter approaches (Kira and Rendell, 1992), wrapper approaches (Kohavi and John, 1997), and embedded approaches (Weston et al., 2003). Filter approaches rely on the statistical properties of features and remain detached from model training. In contrast, wrapper techniques frame feature selection as a search problem, assessing subsets based on learner accuracy. Embedded methods integrate attributes during model learning. Recent advances in deep-learning technology have yielded novel selection methods. Methods utilizing autoencoders (Han et al., 2018) and sparse matrix regression approaches (Hou et al., 2017) have demonstrated notable efficacy. Deep learning methods that automatically learn high-dimensional features continue to gain research attention. However, a tradeoff between interpretability and computational efficiency persists. In this study, we selected GAs (Sukhija et al., 2016) to eliminate redundant and invalid features from the complete set of facial geometric features. This GA selection optimized the subset of MA geometric features for the facial beauty evaluation task. By integrating prior MA constraints and designing a multi-objective fitness function, we reduced computational burden and parameter sensitivity, while ensuring that the selected features met clinical MA standards.

### 2.2 Facial landmark model

Facial landmark models are essential technologies in computer vision that analyze facial structure, estimate head pose, and interpret semantic information by identifying key landmarks such as the corners of the eyes, the tip of the nose, and the edges of the mouth. Facial landmarks can be localized through various approaches, including the active shape model (ASM) (Milborrow and Nicolls, 2008), active appearance model (AAM) (Cootes et al., 1998), and cascade regression (Tong and Zhou, 2021). The ASM uses statistical shape models for landmark detection. This approach requires numerous training images to be manually labeled for the establishment of a statistical model of facial shapes. It encompasses both average shape and patterns of shape variations. During detection, the ASM precisely localizes facial landmarks by matching the statistical shape model to the target image through iterative optimization. As an extension of the ASM, the AAM adds texture information to shape data, thereby describing facial appearance more comprehensively. Cascade regression is a rapid facial landmark detection method that utilizes regression analysis. This technique refines the prediction results for facial landmarks by training a series of regressors. In the deep-learning-driven stage, facial landmark detection methods based on heatmap regression models (Zou et al., 2019) generate likelihood heatmaps of landmark positions using convolutional neural networks (CNNs) and directly regress the coordinates. We adopted a cascade regression architecture with a progressive prediction mechanism to train our MA facial landmark model because CNNs are sensitive to the scale of the training data and prone to overfitting with small sample sizes.

### 2.3 Facial beauty evaluation

Early studies on facial beauty assessment heavily relied on machine learning. For example, Chen et al. (2016) introduced a data-driven framework for facial aesthetic analysis that comprises three core modules: prediction, retrieval, and manipulation. In the prediction module, researchers combined multiple low-level facial representations with high-level features to generate feature vectors. They then optimized the feature set using systematic feature selection. Experimental results revealed that the model built using the refined feature set surpassed previous leading methods. Iyer et al. (2021) conducted a comparative analysis of four machine-learning algorithms (K-nearest neighbor (KNN), linear regression, random forest, and artificial neural networks) for facial beauty prediction. Their findings indicated that the KNN model achieved the best performance when facial landmarks were incorporated with texture, shape, and color features. Similarly, Eisenthal et al. (2006) used multiple machine-learning techniques to predict facial attractiveness and demonstrated strong agreement (*r* = 0.650) between the trained predictor and average human scores.

Given the remarkable performance of deep learning in facial beauty assessment, Xie et al. (2015) used a CNN model for facial attractiveness prediction using the SCUT-FBP dataset and achieved an optimal Pearson’s correlation (PC) coefficient of 0.8187, underscoring the potent capability of the CNN model to recognize facial beauty. Xu et al. (2017) introduced a psychology-inspired CNN (PI-CNN) for automatically predicting facial beauty. Zhai et al. (2019) introduced BeautyNet, a model that integrates multiscale CNNs and transfer learning for unconstrained facial beauty prediction. Lin et al. (2019) developed a relative ranking regression method based on R^3^CNN for facial beauty prediction. R^3^CNN integrates facial aesthetic relative ranking information to boost the performance of facial beauty prediction.

Although deep-learning methods have demonstrated high accuracy, explicitly identifying the factors that contribute to facial beauty evaluations remains challenging. Consequently, traditional machine-learning methods offer greater interpretability and can utilize visible features to assess facial beauty. For example, Mao et al. (2010), Zhang et al. (2018), and Peng et al. (2023) extracted 17, 42, and 115 geometric features from facial data in their respective studies. In the present study, fundamental facial features were consolidated from previous research, and new clinician-driven descriptors were added to construct a robust facial landmark model for clinical evaluation.

## 3 Methodology

**Figure 1:**
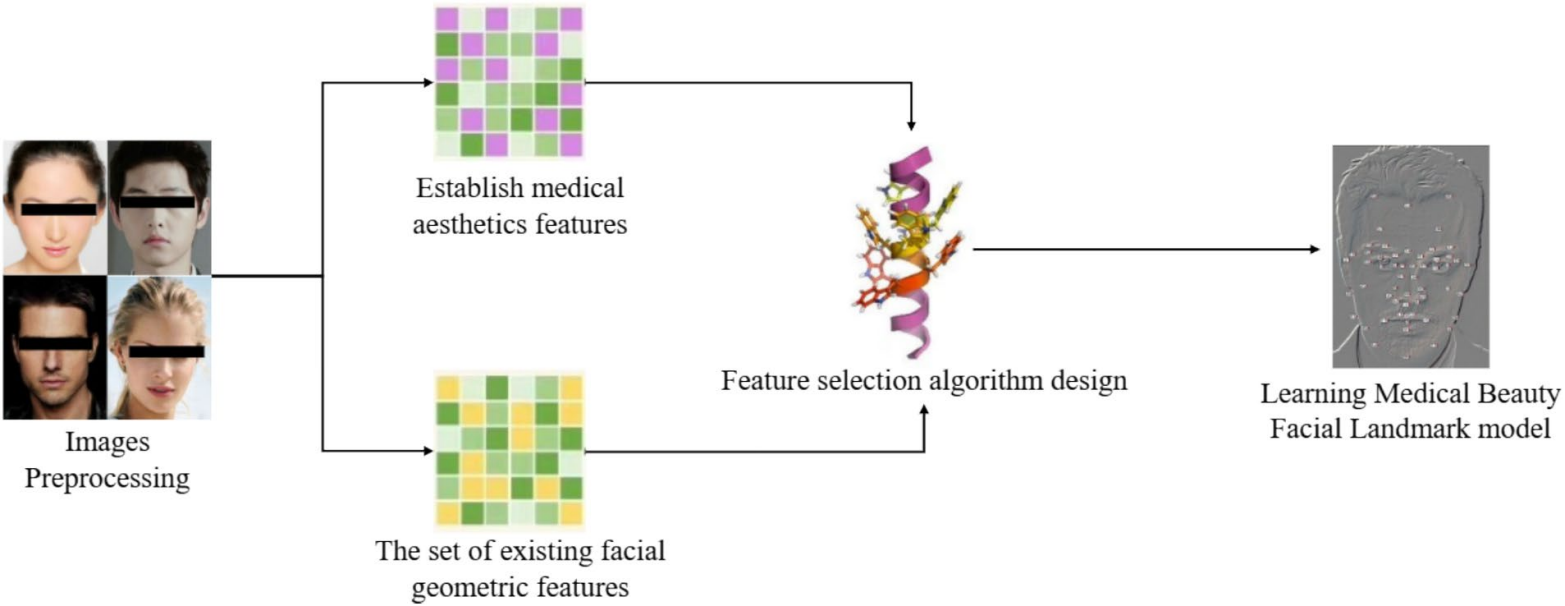
Flowchart for learning a medical aesthetic facial landmark model.

Facial aesthetic prediction is a quantitative problem of subjective perception. Therefore, we must first clarify where the “standard of beauty” learned by the model originates from and convert it into a computable numerical form. The SCUT-FBP dataset, the SCUT-FBP5500 dataset, and the CFD dataset, which provide strictly collected and labeled aesthetic truths based on public consensus, are the reliable starting points for this study. At the same time, their openness ensures the reproducibility of the research. The standard, this method can effectively smooth out individual preference differences and obtain a stable estimation of the mainstream aesthetic of society. The overall workflow of the proposed MA facial landmark model is illustrated in Figure 1. First, a comprehensive set of aesthetic medical facial features was constructed. This set combines basic geometric features derived from prior literature and the novel MA features introduced in this study. Then, a 72-keypoint model was introduced that encapsulates these features. To determine the precise positions of these facial key points, we trained a detection model using a cascade regression algorithm. A feature selection method was then developed that integrates prior MA constraints with a multi-objective GA to select an optimal subset of MA facial features. Finally, we used the subset chosen to train a facial landmark model for the MA facial beauty evaluation.

### 3.1 Compilation of facial features for MAs

**Figure 2:**
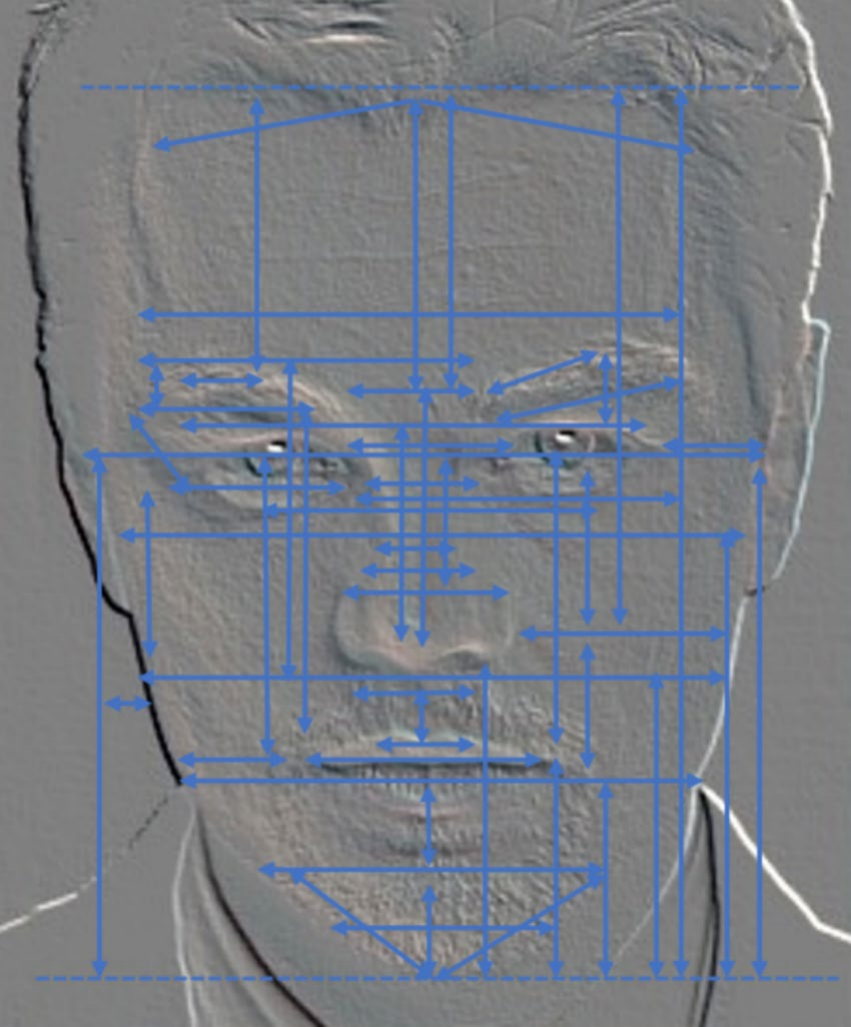
Combination of geometric features extracted from previous studies.

**Figure 3:**
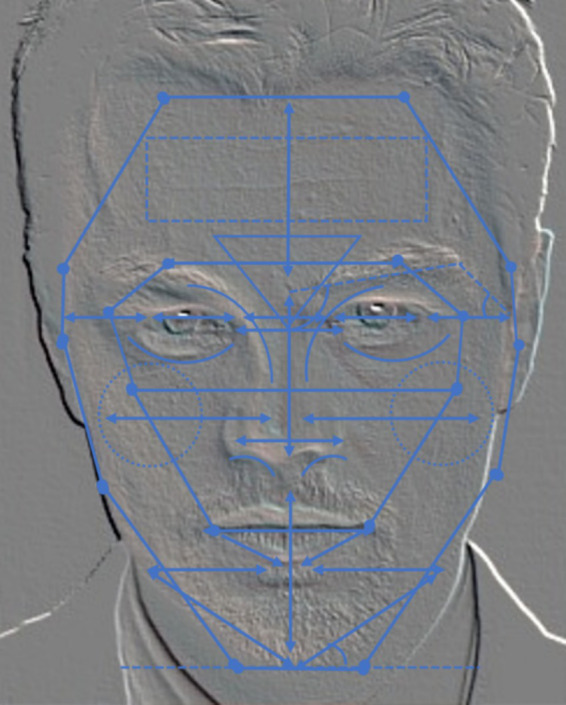
Geometric features in medical aesthetics.

Different facial features provide a fundamental basis for clinicians to develop personalized treatment plans. Previous studies have chiefly characterized facial features by essential geometric attributes, including distance, ratio, and angle. Distance features delineate the spatial relationships between facial contours and specific landmarks, ratio features quantify the proportional relationships between distances, and angle features capture the angles between key facial structures. Building on prior research in machine-learning-based facial beauty analysis (Mao et al., 2010; Peng et al., 2023; Zhang et al., 2016a), we compiled a set of relevant geometric features that were illustrated on a facial image (Figure 2). The double-arrowed line indicates the distance between two key points, while the dotted line defines the range of lengths for measuring the distance. This visualization revealed that traditional geometric features primarily describe inter-organ distances and angles but overlook more nuanced anatomical details. To address this limitation, we constructed a facial MA feature system based on clinical practice, thus emphasizing a clear anatomical orientation suitable for clinical evaluation and cosmetic surgery planning. As shown in Figure 3, the proposed MA features reflect facial harmony. Ablation experiments verified their impact on the assessment of facial attractiveness. Additionally, we added 20 novel MA features (Table 1), including angles and ratios tailored to key facial regions such as eyebrows, eyes, nose, lips, and chin. These features follow established MA principles. The MA feature set represents clinical aesthetic criteria through specialized formulations. For zygomatic symmetry assessment, we introduce an asymmetry index as Equation 1:


$$A_{z}=\frac{1}{n}\sum_{i=1}^{n}\frac{|d(L_{i},R_{i})|}{\max(d(L_{i},C),d(R_{i},C))}$$ (1)

Where $L_{i}$ and $R_{i}$ denote corresponding left and right zygomatic landmarks, C represents the facial midline, and d(·) calculates Euclidean distance. Equation 2 shows the nasolabial angle $\theta_{\mathrm{nl}}$ combines both angular measurement and proportionality:


$$\theta_{\mathrm{nl}}=\arctan(\frac{y_{\mathrm{sn}}-y_{\mathrm{ls}}}{x_{\mathrm{sn}}-x_{\mathrm{ls}}})-\arctan(\frac{y_{\mathrm{sn}}-y_{c}}{x_{\mathrm{sn}}-x_{c}})$$ (2)

With *sn* (subnasale), *ls* (labiale superius), and *c* (columella) landmarks. Equation 3 shows that the three vertical facial divisions are quantified through proportional indices:


$$P_{v}=\frac{\max(h_{\text{upper}},h_{\text{middle}},h_{\text{lower}})}{\min(h_{\text{upper}},h_{\text{middle}},h_{\text{lower}})}$$ (3)

Where h denotes the height of each facial third. We integrated 115 reported geometric features (distance, ratio, and angle) with these 20 innovative anatomically oriented features to develop a comprehensive MA facial feature set comprising 135 features. We performed rigorous cross-validation with a panel of six board-certified aesthetic surgeons (three plastic and three dermatologic) to ensure the medical validity of this feature set. For inter-rater reliability, the intraclass correlation coefficient was calculated at 0.87 (95% confidence interval [CI] 0.82–0.91). Compared with existing approaches, our feature set offers greater interpretability and clinical applicability to facial beauty evaluation for MA purposes. This finding supports precise medical applications such as cosmetic surgery planning and facial rejuvenation assessment.

**Table 1:** Facial MA features and quantitative indicators (overall proportions).

Facial features	Quantitative indicators (reference range)	MA applications
Overall proportions		
1. Ratio of facial width to facial height	Golden ratio ≈1:1.618 (±5%)	Overall coordination assessment and contour surgery design
2. Proportion of the three chambers	Ideal ratio 1:1:1 (±10% allowed)	Lower middle face fillers or osteotomy adjustments
3. Five-eye proportions	Monocular width ≈ face width/5 (error < ±8%)	Hypertelorism/narrow correction (opening the corner of the eye)
Eye area		
4. Proportion of the horizontal width of the eye fissure to its vertical height	Ideal ratio ≈ 3:1 (females may be slightly higher)	Double eyelid surgery width design
5. Binocular spacing ratio	Intercanthal distance ≈ face width/5 (error < ±5%)	Epicanthoplasty (opening the inner canthus)
6. Ptosis index	Occlusion ≤ 1/4 (> 1/3 required levator correction)	Correction of blepharoptosis
Nasal area		
7. Alar width ratio	Ideal ratio ≈ 1:1 (error < ±10%)	Alar constriction
8. Nasofrontal angle	120°–135°	Nose root filling
9. Midline deviation of the nasal bridge	Offset ≤ 1 mm	Corrective surgery for the nose
Lip area		
10. Lip width ratio	Ideal ratio ≈ 1.5:1 (±15%)	Thin/thin lip fillers
11. Philtrum length ratio	Ideal ratio ≈ 1:3 (slightly shorter for women)	Philtrum shortening
12. Proportion of upper to lower lip thickness	Ideal ratio ≈ 1:1.6 (slightly fuller lower lip for women)	Lip shape adjustment (hyaluronic acid injection)
Jawline		
13. Jaw angle	110°–130°	Mandibular angle osteotomy/dermabrasion
14. Chin width ratio	Chin width ≈ 2/3 (±10%) of the distance between mandibular angles	Chin osteotomies or implants
Symmetry		
15. Right and left zygomatic symmetry	Difference is ≤ 1 mm	Zygomatic asymmetry correction surgery
16. Brow peak symmetry	Difference is ≤ 1.5 mm	Eyebrow lifting/eyebrow arch filling
17. Horizontal angle of the mouth offset	Difference is ≤ 1 mm	Mouth lift (to improve a crooked mouth)
16. Brow peak symmetry	Difference is ≤ 1.5 mm	Eyebrow lifting/eyebrow arch filling
17. Horizontal angle of the mouth offset	Difference is ≤ 1 mm	Mouth lift (to improve a crooked mouth)
Local coordination		
18. Zygomatic-chin width ratio	Ideal ratio ≈ 1.2:1 (slightly higher for women)	Facial contour integrated design (“inverted triangle face” shaping)
19. Eye-to-nose ratio	Ideal ratio ≈ 1:1 (±10%)	Comprehensive eye and nose plastic surgery
20. Frontotemporal ratio	Ideal ratio ≈ 0.9:1 (±5%)	Temple fillers/zygomatic thrusts

**Figure 4:**
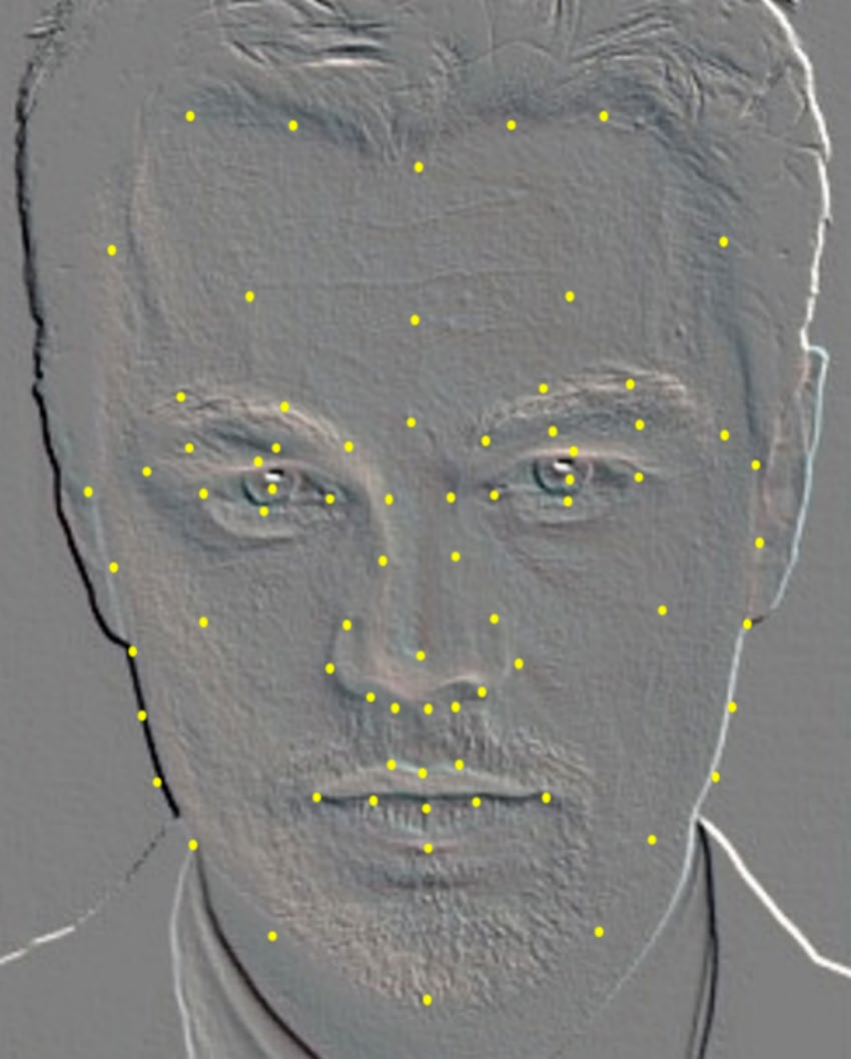
Medical aesthetic facial landmark model (72 points).

To accurately extract the systematic set of 135 features for MAs, a specialized facial landmark detection model was required. Although existing facial landmark detection models effectively capture the basic geometric features of the face, they lack MA priors and are therefore unsuitable for constructing our feature set. Consequently, we propose a facial landmark model that incorporates MA knowledge with 72 labeled points (Figure 4).

### 3.2 MA facial landmark detection and feature extraction

We built a 72-point MA facial landmark detection model for the SCUT-FBP5500 dataset using a cascaded pose regression (CPR) framework. The core idea of this algorithm involves learning *V* linear regressors from pairwise positional information (x- and y-coordinates) of manually annotated facial keypoints. Each regressor refines shape features by combining the output of the previous regressor with the input face image, progressively enhancing the accuracy of keypoint location predictions. This algorithm uses a progressive prediction approach that iteratively adjusts the positions of landmarks. Given an input face image X, the landmark positions are refined iteratively by Equation 4:


$$S_{v}=S_{v-1}+\alpha_{v}\cdot R_{v}(X,S_{v-1})$$ (4)

Where $\;S_{v}$ denotes the landmark estimation at the *v*-th stage, $R_{v}$ represents the *v*-th stage regressor, and $\alpha_{v}$ represents the learning rate decay coefficient, set to 0.8. The initial shape $S_{0}$ is set to the mean shape, and the final $S_{v}$ represents the keypoint locations for the given facial image X.

### 3.3 Feature selection algorithm

Using the MA facial landmark model, we extracted a 135-dimensional feature vector for each face from the SCUT-FBP5500 dataset. However, certain features were redundant or insignificant in facial beauty evaluation because of overlapping contributions. To address this issue, we deployed a feature selection process that incorporates prior MA constraints with multi-objective GAs.

The feature selection process uses a modified GA that enforces MA constraints through three mechanisms. Chromosomes are represented using binary encoding $x\in {\left\{0,1\right\}}^{135}$ , where $x_{i}=1$ indicates feature selection. During the population initialization stage, we applied a prior-informed strategy that incorporates prior MA knowledge to initialize all individuals in the population, rather than relying on random initialization. This approach reduced the unproductive search space and accelerated convergence by ensuring that clinically significant features were retained. Specifically, given *N* total features indexed as {1, 2,…, *N*}, initialization ensured that these prior-informed features were embedded in each chromosome. Prior knowledge in MAs comprised 20 specific features, indexed by the set $S=\{s_{1},s_{2},\ldots ,s_{M}\}$ , where S⊆{1,2,…,N} . Each chromosome was encoded as $x=[x_{1},x_{2},\ldots ,x_{N}]$ , with $x_{i}\in \{0,1\}$ . The chromosome procedure for each $x_{i}$ is defined as Equation 5:


$$x_{i}=\{\begin{array}{cc} 1, & \text{if}\;i\in S\\ \text{Bernoulli}(p), & \text{otherwise} \end{array}$$ (5)

The gene of i∈S is directly assigned a value of 1 to ensure that prior knowledge is retained. p ∈ [0,1] denotes the random selection probability for other features, which was predefined as *p* = 0.6 in this context.

For the multi-objective optimization task of feature selection, we defined a dual-criterion fitness function to evaluate performance. This function targets high performance and minimizes the number of selected features. Equation 6 shows that the fitness function comprises two components and is designed to be maximized. Here, PC(x) is the evaluation metric for the beauty score performance, and *N* and *S* denote the dimensions of the original and selected feature subsets, respectively.


$$F(x)=\alpha \cdot \mathrm{PC}(x)+\beta \cdot (1-\frac{S}{N})$$ (6)

Equation 7 shows that during the mutation operation, a targeted mutation strategy was devised for the key features of MAs:


$$x_{i}^{\mathrm{new}}=\{\begin{array}{cc} 1-x_{i} & \text{if}\;i\notin S\;\text{and}\;\mathrm{rand}()<p_{m}\\ 1 & \text{if}\;i\in S,\\ x_{i} & \text{otherwise} \end{array}$$ (7)

ALGORITHM 1 Pseudocode of the feature selection algorithm.
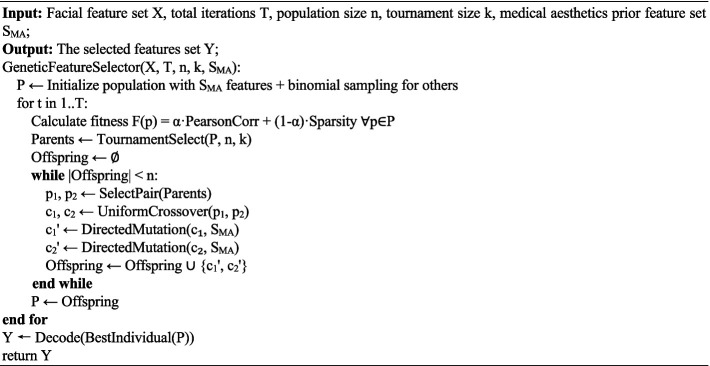

### 3.4 MA facial landmark model

**Figure 5:**
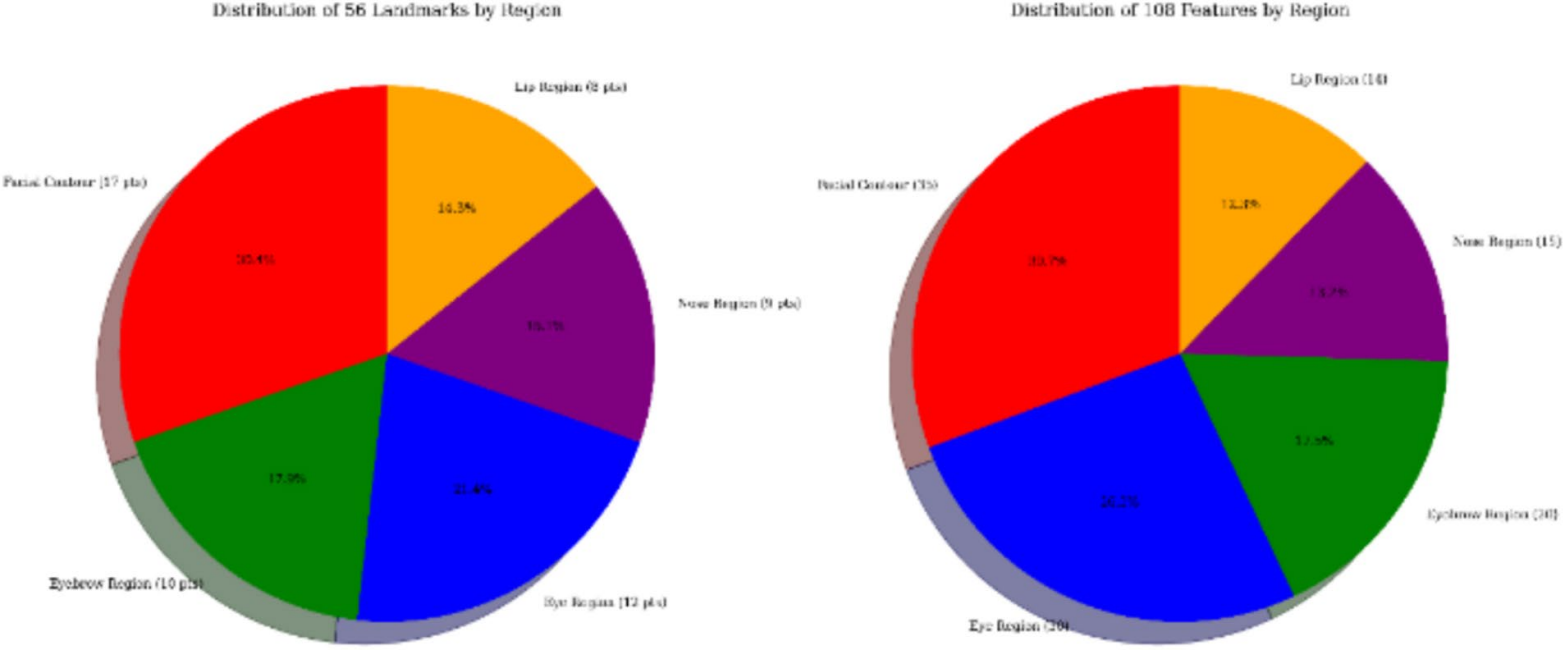
Fifty-six landmarks and 108 features distribution.

After feature selection, a 108-dimensional feature subset was extracted from the initially constructed 135-dimensional feature set, all of which contributed to facial beauty assessment. The details are summarized in Figure 5.

**Figure 6:**
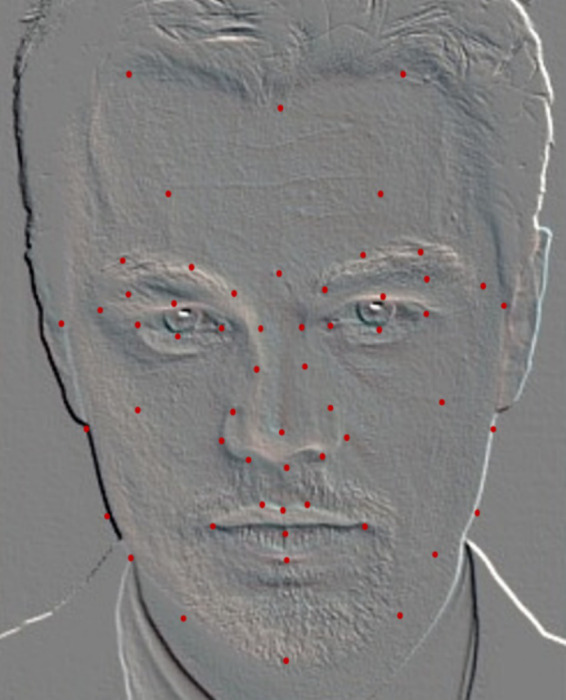
Optimized medical aesthetic facial landmark model (56 points).

In the initial phase of feature-space construction, we built an MA facial model comprising 72 facial landmark points based on the 135-dimensional geometric features. By incorporating prior MA knowledge and using an enhanced multi-objective GA for feature selection, we identified a 108-dimensional feature subset with high explanatory power. This subset was aligned with an optimized model constructed from 56 key aesthetic medical points (Figure 6). The new MA facial landmark model was tailored to evaluate facial beauty in the context of MAs, where each point contributed relevant features to the perception of facial beauty. Experimental results showed that our model enhanced the interpretability of critical facial regions. Local facial proportions and contour characteristics contributed most significantly to the assessment. By integrating prior MA constraints and enabling automatic alignment of machine-learning feature importance, this model established an interpretable computational aesthetic framework for digital assessment in MAs.

## 4 Experiment

### 4.1 Dataset

The SCUT-FBP database (Xie et al., 2015), developed by SCUT, contains 500 frontal facial images of young Asian females. Each image has a beauty score (1–5, with higher indicating greater beauty) averaged from 70 volunteer ratings. Images in this dataset vary in resolution.

In contrast, SCUT-FBP5500 (Liang et al., 2018), also from SCUT, is a larger database featuring 5,500,350 × 350-pixel color frontal facial images. This dataset is more diverse, including 2,000 Asian males, 2,000 Asian females, 750 Caucasian males, and 750 Caucasian females. Beauty scores (5-point scale) were assigned by 60 raters viewing photos in random order, with a consistent standard deviation of 0.6–0.7 between individual and final scores.

**Figure 7:**
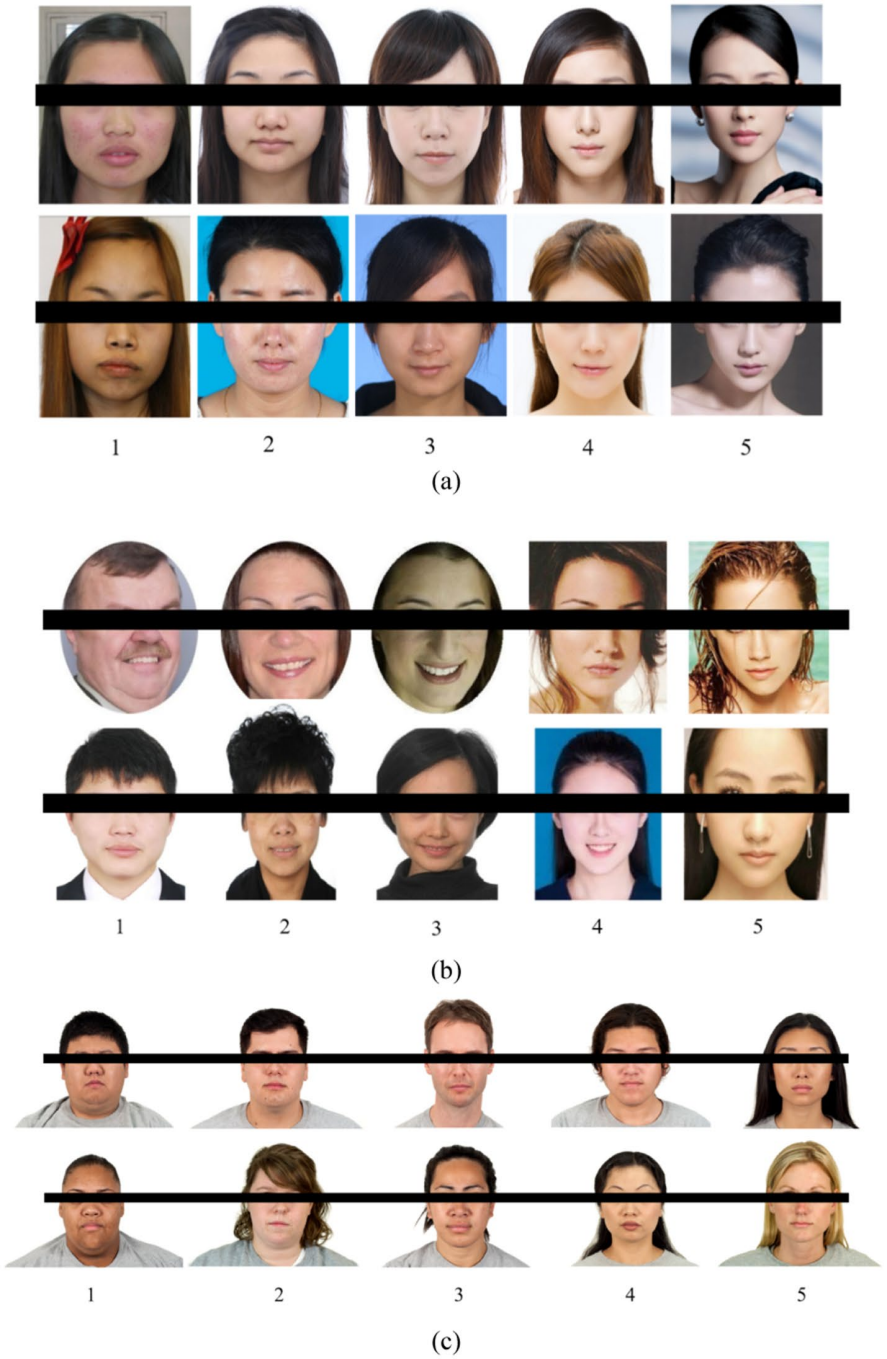
Image samples from the three facial beauty databases: **(a)** SCUT-FBP, **(b)** SCUT-FBP5500, and **(c)** CFD.

The CFD (Ma et al., 2015) contains images of 597 distinct individuals from the United States (U. S.), encompassing self-identified Asian, Black, Latino, and white males and females. All models display a neutral facial expression, with corresponding norming data available. Subjective beauty ratings, ranging from 1 to 5, were also derived from U. S.-based raters. Figure 7 shows image examples from the three datasets.

Experiments were conducted on NVIDIA RTX 3090 GPUs (24 GB VRAM) using the PyTorch framework. For a fair comparison, standard dataset splits were used: 60% for training, 20% for validation, and 20% for testing. Data augmentation techniques included random horizontal flipping (±10° rotation), color jitter (brightness, contrast, and saturation all set to 0.2), and Gaussian blur (*σ* = 0.1). Before landmark detection, we conducted image conversion, rotation, and alignment procedures to ensure the precise spatial registration of facial images, thereby enhancing the consistency and reliability of subsequent analysis.

**Figure 8:**
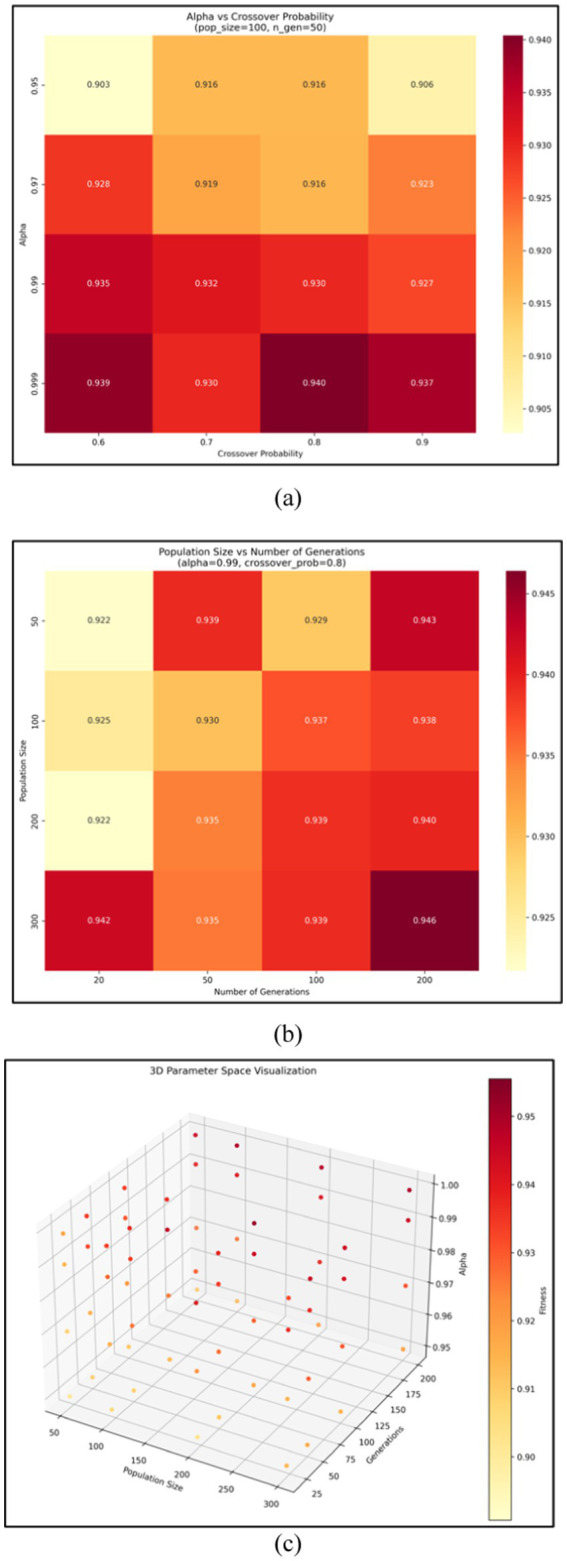
Visualization of the parameter selection process.

Parameters were set to preserve critical MA features while allowing the exploration of geometric features (Figure 8 and Table 2).

**Table 2:** Algorithm parameter settings.

Parameter	Value
Population size	200
Generations	200
Alpha value	0.999
Beta value	0.001
Crossover probability	0.8
Tournament size	3

### 4.2 Evaluation method

In facial beauty evaluation research, three evaluation criteria are frequently used to assess the performance of facial beauty scoring models, namely: Pearson’s correlation coefficient (PC), mean absolute error (MAE), and root mean square error (RMSE). The metric PC measures the association between predicted ratings and ground-truth labels as Equation 8:


$$\mathrm{PC}=\frac{\sum_{i=1}^{n}\;(a_{i}-\bar{a})(b_{i}-\bar{b})}{\sqrt{\sum_{i=1}^{n}\;{\left(a_{i}-\bar{a}\right)}^{2}\sum_{i=1}^{n}\;{\left(b_{i}-\bar{b}\right)}^{2}}}$$ (8)

MAE represents the average absolute discrepancy between the predicted and true values as Equation 9:


$$\mathrm{MAE}=\frac{1}{n}\sum_{i=1}^{n}|a_{i}-b_{i}|$$ (9)

where *a* and *b* represent the predicted evaluation value and the actual label value, respectively, for the *i*-th face image, and *n* denotes the total number of samples in the dataset.

RMSE was used to measure the error between the predicted evaluation value *a* and the true label value *b* as Equation 10:


$$\text{RMSE}=\sqrt{\frac{1}{n}\sum_{i=1}^{n}\;{\left(a_{i}-b_{i}\right)}^{2}}$$ (10)

### 4.3 Evaluation of feature selection methods

In MA feature engineering, the efficiency of feature selection algorithms directly affects both the interpretability and speed of the model. To assess the advantage of the proposed multi-objective GAs based on prior knowledge of MAs, we constructed a comparative framework encompassing four significant types of feature selection methods:

1. Classic ensemble learning (random forest) (Hasan et al., 2016).
2. Linear regularization (lasso regression) (Fonti and Belitser, 2017).
3. Swarm intelligence algorithm (GWO) (Mirjalili et al., 2014) and (BWOA) (Tawhid and Ibrahim, 2020).
4. Multi-objective feature selection method (MOFS) (Mlakar et al., 2017).
5. A hybrid CNN (AestheticNet) (Danner et al., 2022).

The results obtained from the SCUT-FBP, SCUT-FBP5500, and CFD test datasets are listed in Table 3. Our method achieved the highest PC (0.8216) and lowest errors (MAE: 0.2638, RMSE: 0.3743) while selecting only 108 features, demonstrating greater efficiency and accuracy. This comparative experiment highlights the necessity of intelligent algorithms guided by domain-specific medical knowledge during feature selection tasks.

**Table 3:** Comparison of different feature selection methods.

Method	Feature dimensions	PC	MAE	RMSE
Random forest	256	0.7445	0.4165	0.4832
Lasso	198	0.7621	0.4041	0.4545
GWO	188	0.8012	0.3467	0.4038
BWOA	135	0.7828	0.3695	0.4180
MOFS		0.8046	0.3252	0.3768
AestheticNet		0.8124	0.3024	0.3891
Ours	108	0.8216	0.2638	0.3743

**Figure 9:**
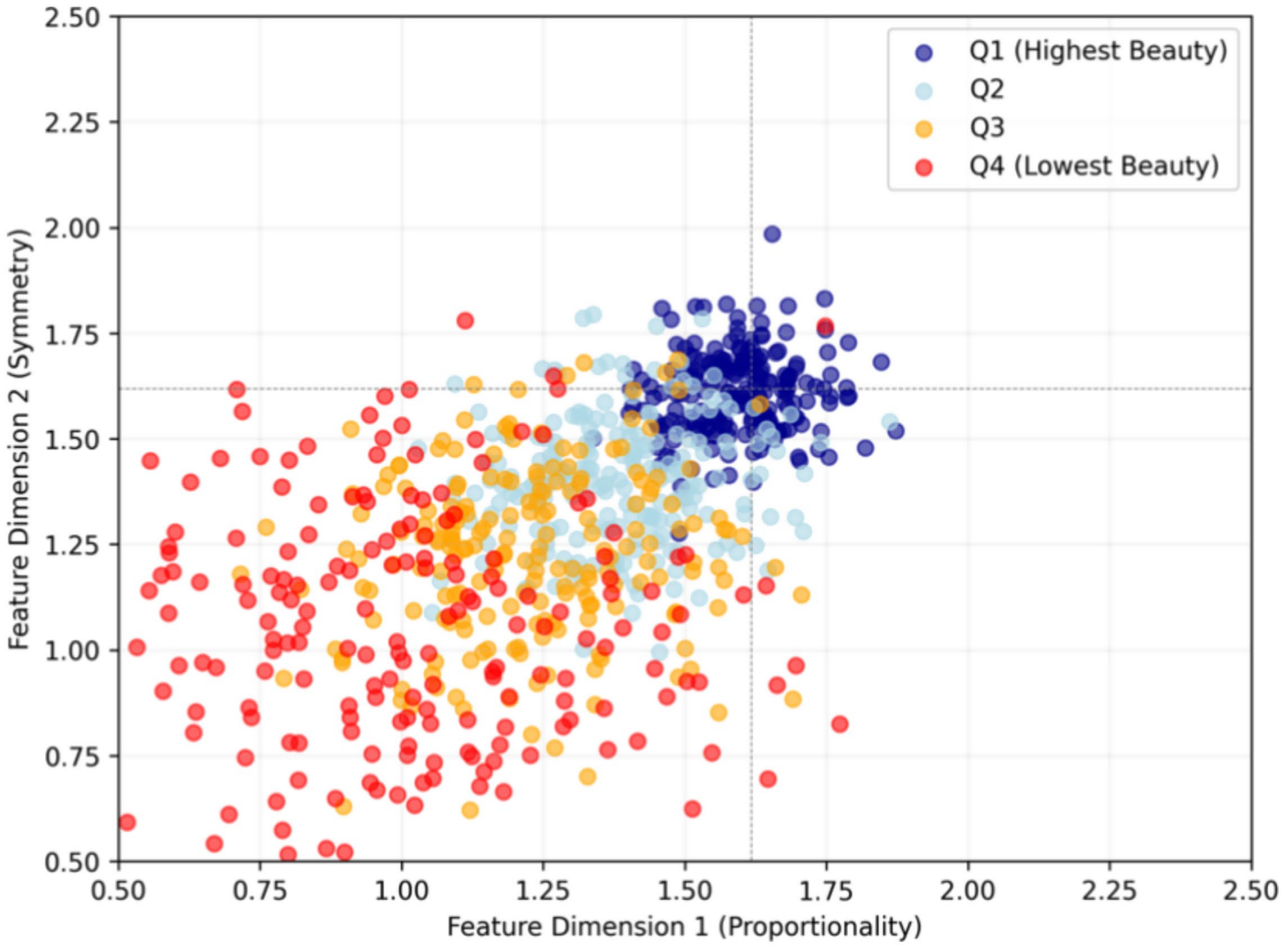
Projection of facial features into 2D space, colored by beauty score quartiles.

The selected feature subsets reveal medically meaningful patterns. Figure 9 shows the clear separation of high- and low-beauty score faces in the reduced feature space, aligning with medically relevant dimensions.

### 4.4 Evaluation of the medical beauty facial landmark model

**Figure 10:**
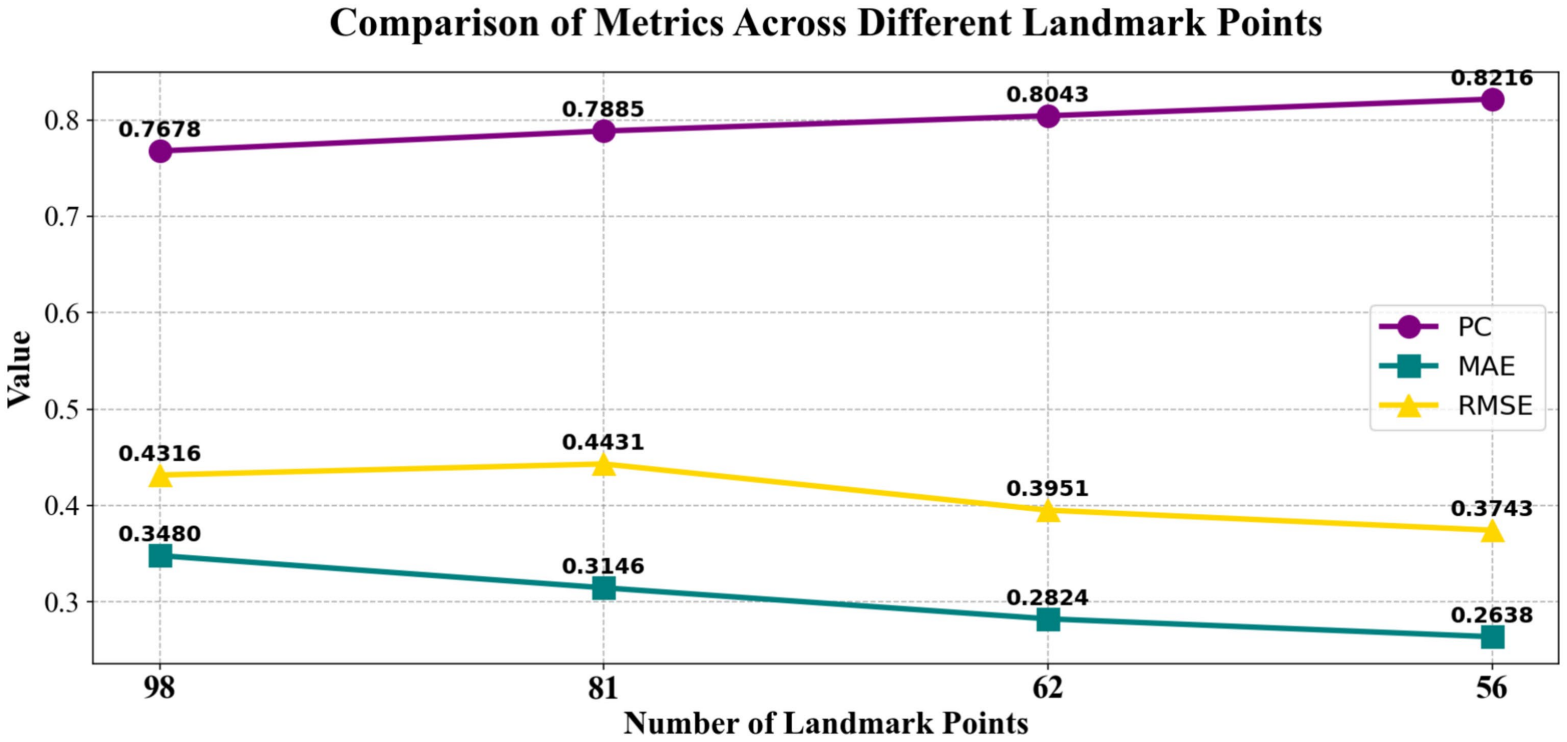
Comparison results of different facial landmark models.

Traditional quantitative research on facial aesthetics lacks clearly defined keypoint selection criteria. Existing facial landmark systems, such as the 98-point (Zhang et al., 2016b), 81-point (Zhao et al., 2020), and 62-point (Peng et al., 2023) models, primarily focus on facial recognition tasks and overlook the specific MA requirements. Although commonly used basic positioning points, such as the nasal tip and brow peak, describe facial contours, they overlook aesthetic feature areas vital to clinical practice. These areas include key anataomical markers such as the turning point of the nasolabial angle and the highest point of the zygomatic bone. To validate the advantage of the proposed model, we evaluated the performance of various facial keypoint models from previous studies using the SCUT-FBP, SCUT-FBP5500, and CFD test datasets. As shown in the summary of the findings in Figure 10, reducing landmarks from 72 to 56 points improved PC by 2.13%, because non-critical landmarks introduced noise.

### 4.5 Ablation studies

To validate the efficacy of the constructed 20-dimensional MA feature set for evaluating facial beauty, we designed a stepwise ablation framework. Using the control variable method, five groups of feature combinations were built: the baseline group *N* (comprising only traditional geometric features) and the experimental groups S1–S4, which gradually incorporated subsets of MA features. The contribution of each feature combination to the model performance was assessed through stratified cross-validation. Twenty MA features formed four subsets, S_1_, S_2_, S_3_, and S_4_. Experimental results (Table 4) demonstrate that across five different feature combinations, the inclusion of MA features improved the discriminative power of the model. As the feature set expanded from *N* to S_4_, the PC increased from 0.7082 to 0.8216, achieving 16% improvement. This finding suggests that the incremental addition of MA features and the proposed feature set progressively boosted the ability of the model to evaluate facial beauty.

**Table 4:** Ablation studies for different combinations of features.

Features used	PC	MAE	RMSE
N	0.7082	0.4121	0.4874
N + S1	0.7245	0.3816	0.4762
N + S1 + S2	0.7831	0.3247	0.4239
N + S1 + S2 + S3	0.8053	0.2895	0.3918
Full (N + S₁–S₄)	0.8216	0.2638	0.3743

**Figure 11:**
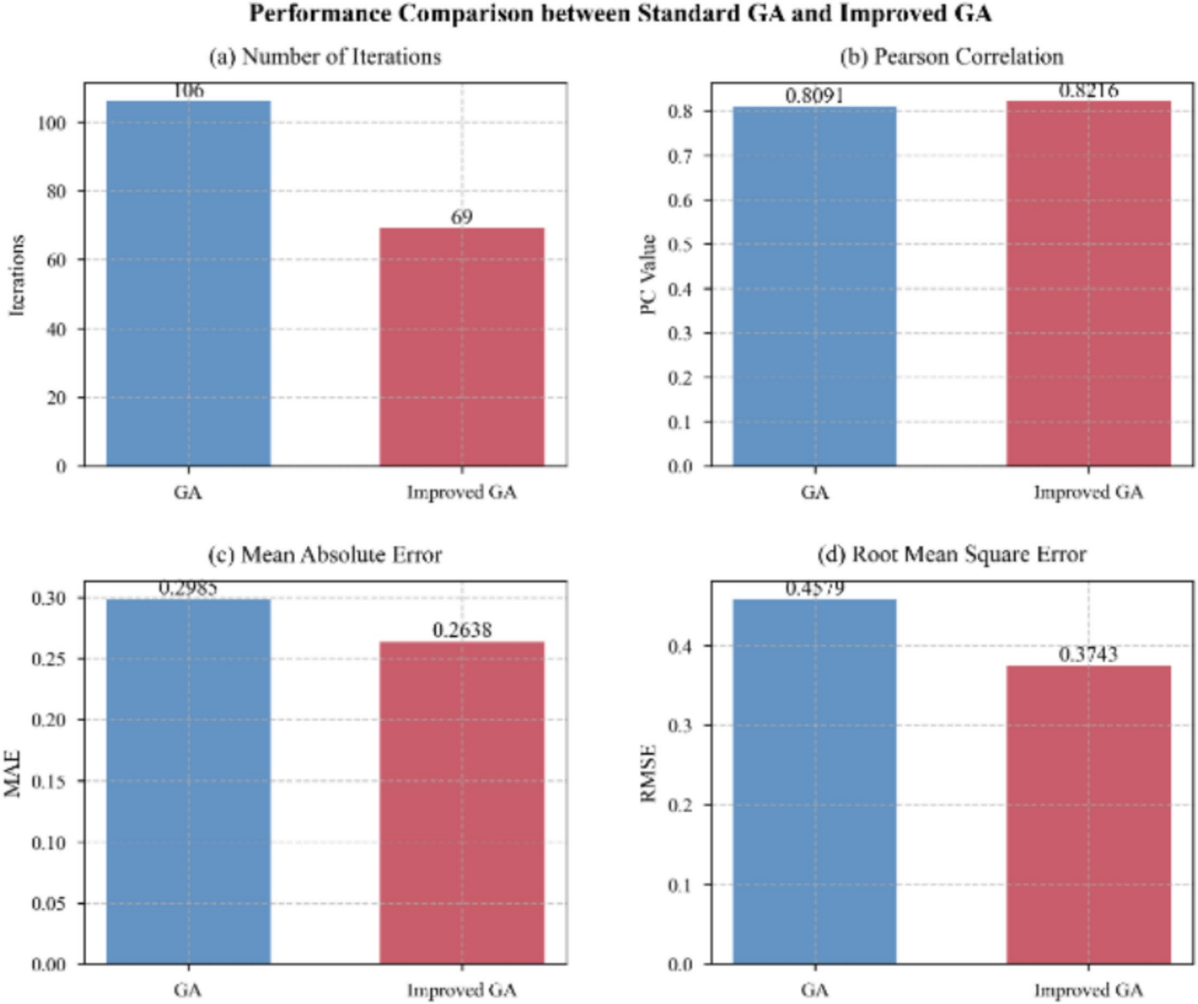
Performance comparison of GAs and improved GAs.

Additionally, to assess the effectiveness of the feature selection method, we used an enhanced GA that incorporated MA features as a mandatory component for initializing all individuals in the population. We also applied the directional mutation strategy. This approach preserved core MA indicators during evolution while enabling thorough exploration of non-critical features. This approach speeded convergence and enhanced the solution quality. Ablation studies were conducted to confirm the effectiveness of this approach (Figure 11).

## 5 Discussion

Our approach advances medical prior knowledge from a posterior explanatory element to a structural prior that guides the algorithm’s search process. Unlike general-purpose methods such as Lasso and random forest, it offers a specialized solution designed specifically for medical feature selection. In contrast to unbiased optimizers such as GWO, it functions as a guided intelligent navigator. Compared to the “black box” nature of deep learning, it serves as a transparent and trustworthy decision-making advisor. Although the proposed method demonstrates improved accuracy and clinical relevance, several limitations remain. First, the performance of the model depends on the quality of initial landmark detection, which may degrade on occluded or non-frontal faces. Although the CPR framework incorporates iterative refinement, extreme head rotations (> 30°) break the assumptions behind many MA features (for example, bilateral symmetry metrics). Second, the current feature set emphasizes geometry, potentially overlooking texture-based attributes such as skin smoothness and pigmentation uniformity that contribute to aesthetic judgments. Third, the integration of clinical standards into computational models raises ethical questions about representation across cultures. For instance, the “three-temple-five-eye” proportion reflects East Asian beauty norms and may not transfer to other ethnic groups. Similarly, the nasofrontal angle range originated in Caucasian populations and could misrepresent aesthetic ideals for individuals with different nasal morphologies. The interpretability of MA-selected features enables direct translation to clinical workflows, such as preoperative planning for orthognathic surgery or rhinoplasty. Surgeons could use the model’s symmetry scores and angular measurements to quantify asymmetries or deviations from ideal proportions, supplementing qualitative assessments. However, real-world deployment requires addressing two challenges: (1) integrating 3D facial scans to capture volumetric aesthetics beyond 2D projections and (2) developing interactive tools that allow clinicians to adjust feature weights based on patient-specific goals. Future research should develop dedicated models for specific cultures or groups rather than pursuing a “universal” model that applies to everyone. It can explore the reinforcement learning framework to achieve personalized aesthetic evaluation, where the model adapts to individual preferences through iterative feedback.

## 6 Conclusion

This study aimed to address core challenges at the intersection of computer vision and MAs by creating a robust quantitative framework for assessing facial beauty. First, we systematically collected traditional facial geometric features from the literature and constructed a multiscale facial feature set relevant to MAs. This comprehensive feature set captures the contours and fine details of various facial regions aligned with clinical aesthetic principles.

We then used a multi-objective GA informed by medical-aesthetics expertise to select the most impactful facial features for assessing facial beauty. Based on the optimized feature subset, we developed a facial landmark model grounded in MAs with 56 keypoints. This model achieved fast inference and required fewer training samples compared to conventional approaches. Experimental results demonstrated that the optimized key point model, along with its corresponding medical-aesthetics geometric features, significantly outperformed machine-learning models that rely solely on traditional facial features. Our research facilitates the real-time assessment of facial beauty and offers practical guidelines for cosmetic surgery and automated facial enhancement applications.

Moreover, the principles established in this study may extend beyond facial beauty assessment to other medical imaging domains where interpretability is paramount. Integrating clinical knowledge with machine-learning optimization advances trustworthy artificial intelligence in healthcare. Despite challenges in generalizing across diverse populations and three-dimensional facial structures, this framework lays crucial groundwork for future research at the intersection of computer vision and MAs.

## Data Availability

The original contributions presented in the study are included in the article/supplementary material, further inquiries can be directed to the corresponding author.
